# Redefining Healthcare With Artificial Intelligence (AI): The Contributions of ChatGPT, Gemini, and Co-pilot

**DOI:** 10.7759/cureus.57795

**Published:** 2024-04-07

**Authors:** Anas Alhur

**Affiliations:** 1 Health Informatics, University of Hail College of Public Health and Health Informatics, Hail, SAU

**Keywords:** natural language processing models, co-pilot, google gemini, medical education, medical research, patient care, health sciences, chatgpt, artificial intelligence (ai)

## Abstract

Artificial Intelligence (AI) in healthcare marks a new era of innovation and efficiency, characterized by the emergence of sophisticated language models such as ChatGPT (OpenAI, San Francisco, CA, USA), Gemini Advanced (Google LLC, Mountain View, CA, USA), and Co-pilot (Microsoft Corp, Redmond, WA, USA). This review explores the transformative impact of these AI technologies on various facets of healthcare, from enhancing patient care and treatment protocols to revolutionizing medical research and tackling intricate health science challenges. ChatGPT, with its advanced natural language processing capabilities, leads the way in providing personalized mental health support and improving chronic condition management. Gemini Advanced extends the boundary of AI in healthcare through data analytics, facilitating early disease detection and supporting medical decision-making. Co-pilot, by integrating seamlessly with healthcare systems, optimizes clinical workflows and encourages a culture of innovation among healthcare professionals.

Additionally, the review highlights the significant contributions of AI in accelerating medical research, particularly in genomics and drug discovery, thus paving the path for personalized medicine and more effective treatments. The pivotal role of AI in epidemiology, especially in managing infectious diseases such as COVID-19, is also emphasized, demonstrating its value in enhancing public health strategies. However, the integration of AI technologies in healthcare comes with challenges. Concerns about data privacy, security, and the need for comprehensive cybersecurity measures are discussed, along with the importance of regulatory compliance and transparent consent management to uphold ethical standards and patient autonomy. The review points out the necessity for seamless integration, interoperability, and the maintenance of AI systems' reliability and accuracy to fully leverage AI's potential in advancing healthcare.

## Introduction and background

In light of recent advancements, the integration of Artificial Intelligence (AI) into health sciences marks a transformative era in healthcare, enhancing everything from service delivery to diagnostics and patient interactions. At the forefront of this shift are sophisticated language models such as ChatGPT, Gemini Advanced, and Co-pilot, each introducing novel capabilities to the healthcare domain. This review examines the roles, strengths, and challenges these AI models bring to health sciences.

From early AI applications to the current sophisticated language models, the progression in healthcare technology is noteworthy. Initial AI implementations laid the groundwork for more advanced machine learning and natural language processing (NLP) applications [1,2]. Today, models like ChatGPT, Gemini Advanced, and Co-pilot represent the forefront of NLP, with their advanced text comprehension and generation capabilities crucial for medical literature analysis and improving patient communications [3,4].

ChatGPT is celebrated for its human-like conversational responses, offering significant opportunities for enhancing patient education and engagement [5]. Gemini Advanced excels in its complex query processing, promising to transform medical information retrieval and research [6]. Co-pilot's potential lies in healthcare software development, simplifying tasks from analyzing medical records to developing health applications [7].

However, the adoption of these AI models in healthcare raises critical ethical and privacy issues, including concerns over algorithmic bias, the transparency of AI decisions, and the protection of patient information [8,9]. This review endeavors to juxtapose ChatGPT, Gemini Advanced, and Co-pilot, illuminating their innovative capabilities in healthcare while addressing the ethical dilemmas their application may entail.

## Review

Revolutionizing healthcare with AI integration

The integration of Artificial Intelligence (AI) technologies, such as ChatGPT, Gemini Advanced, and Co-pilot, is significantly transforming healthcare [1-3]. These technologies offer a broad spectrum of applications, from mental health support and management of chronic conditions to improvements in diagnostic accuracy, making healthcare more accessible, personalized, and efficient [4,5].

To provide a deeper understanding of how each AI model contributes to healthcare, Table 1 presents a comparative analysis, highlighting their core technologies, applications, and ethical considerations [6]. This table illustrates the diverse ways in which these AI models enhance patient care, support medical research, and address healthcare challenges [7].

**Table 1:** Comparative analysis of AI technologies in healthcare This table presents a comparative analysis of three prominent AI models—ChatGPT, Gemini Advanced, and Microsoft Copilot—highlighting their core technologies, applications, interoperability, and ethical considerations within the healthcare sector. It aims to provide a comprehensive overview of how each AI model contributes to enhancing patient care, accelerating medical research, and addressing challenges in health sciences. NLP: Natural language processing

Attribute	ChatGPT	Google Gemini AI	Microsoft Copilot
Core Technology	Advanced NLP and deep learning, part of the GPT family developed by OpenAI, capable of producing human-like text responses.	Advanced AI with the latest Ultra 1.0 model, capable of handling highly complex tasks including coding, logical reasoning, and creative project collaboration.	Advanced AI integrating large language models, designed to enhance productivity across Microsoft 365 applications.
Training Data	Vast corpus of text data up to early 2022, capturing nuances of human language, but with noted limitations in medical-specific content.	Not explicitly mentioned, but likely trained on extensive and diverse datasets, including healthcare-specific data for medical applications.	Likely trained on diverse datasets including healthcare-specific data, given its integration with Microsoft 365 applications used by healthcare organizations.
Healthcare Applications	Virtual patient assistants, clinical decision support, medical recordkeeping, medical education, and patient monitoring.	Potential applications in personalized health coaching, understanding lab and imaging information, aiding in early disease detection, and supporting medical decision-making with an emphasis on multimodality and advanced reasoning.	Streamlining administrative tasks, enhancing clinical documentation, facilitating interdisciplinary communication, and supporting decision-making in patient care.
Diagnostic Support	Limited direct diagnostic capabilities, but potential in supporting diagnostic processes through information provision and analysis.	Enhanced capabilities in analyzing and interpreting medical literature, patient histories, and research data, potentially assisting in diagnosing eye conditions and other healthcare needs.	Potential to assist in compiling and summarizing patient information and medical literature, indirectly supporting diagnostic processes.
Treatment Planning	Can assist in gathering and providing information on treatment options but requires human oversight for accuracy and applicability.	May offer personalized health and wellness features, utilizing individual data for tailored recommendations, similar to a personal health coach.	Can aid in aggregating patient data and medical research to inform treatment plans, though direct treatment planning would require clinician oversight.
Interoperability	Highly adaptable to various healthcare systems through APIs, with applications in enhancing patient-provider communication and healthcare access.	Designed to integrate with Google apps, indicating a high level of adaptability and potential ease of integration into existing healthcare systems and workflows.	Highly interoperable within the Microsoft ecosystem, likely to integrate well with healthcare systems that utilize Microsoft 365, Teams, and other Microsoft applications.
Privacy & Security	Adherence to data protection and privacy protocols is crucial, with ethical considerations for patient data handling.	Emphasis on responsible deployment with extensive ethics and safety testing, including external red-teaming and adherence to Google's AI Principles, ensuring data protection and privacy.	Built with Microsoft's comprehensive data protection and privacy standards, with potential applications in secure patient data handling and communication.
User Interface	User-friendly, conversational interface facilitating easier interaction for patients and healthcare providers.	User-centric design, likely offering an intuitive and seamless experience, particularly with the introduction of a mobile app for on-the-go access.	User-friendly, designed to integrate seamlessly into existing workflows within Microsoft 365 applications, offering a familiar interface for healthcare professionals.
Accuracy & Reliability	Capable of generating coherent responses but may produce inaccuracies; human oversight is essential to verify the information provided.	In blind evaluations, rated as the most preferred chatbot, indicating high accuracy and reliability in understanding and processing complex tasks.	Tailored to enhance productivity and reduce administrative burdens, with a focus on accuracy in data handling and content generation.
Ethical Considerations	Challenges in ensuring non-biased, accurate content; concerns around copyright, medico-legal issues, and the need for transparency and accountability in AI-generated content.	Strong commitment to mitigating issues like unsafe content or bias, with built-in safety features and continuous improvement based on human feedback, reflecting a responsible approach to AI in healthcare.	Adheres to Microsoft's AI Principles, focusing on responsible and ethical AI use, with considerations for safety, privacy, and inclusivity in healthcare applications.

Real-world impact of AI in healthcare

AI technologies like ChatGPT are making strides in mental health care by offering scalable, personalized support. For instance, the Woebot application utilizes ChatGPT to provide cognitive behavioral therapy (CBT) to users with anxiety and depression [8]. Gemini Advanced and Co-pilot, though not primarily conversational, play crucial roles in data analytics and automating routine tasks, thereby optimizing clinical workflows and promoting patient-centered care [9,10]. These technologies collaboratively improve chronic condition management and remote patient monitoring, offering real-time, personalized feedback for conditions like diabetes and heart disease [11]. Their capabilities extend to enhancing diagnostic accuracy and fostering a move towards personalized medicine, particularly in oncology through AI-driven genetic data analysis [12].

Table 2 demonstrates specific use cases of these AI technologies in healthcare settings, supported by real-world examples and references [13]. This table showcases how ChatGPT, Gemini Advanced, and Co-pilot are applied in mental health interventions, predictive analytics, clinical workflow optimization, and more, providing tangible evidence of AI's utility in healthcare [14-16].

**Table 2:** Real-world applications of AI in healthcare AI Technology: Refers to the specific artificial intelligence platform being discussed, including ChatGPT by OpenAI, Gemini Advanced, and Co-pilot by Microsoft. Specific Use Case: The particular scenario or healthcare challenge addressed by the AI technology. Application in Healthcare: Describes how the AI technology is applied within the healthcare setting, detailing its function or role. Real-World Example: Provides an instance where the AI technology has been implemented in a healthcare context, illustrating its practical use and impact.

AI Technology	Specific Use Case	Application in Healthcare	Real-World Example	Reference
ChatGPT	Mental Health Interventions	Deployed in conversational agents to deliver therapy and support for mental health conditions.	Woebot application provides CBT for users experiencing anxiety and depression.	[17]
	Medical Education	Facilitates the training of medical professionals and students through interactive Q&A sessions.	Used in medical education platforms to simulate patient-doctor interactions for educational purposes.	[18]
	Literature Review & Analysis	Assists researchers in summarizing and synthesizing large volumes of medical literature.	Utilized in systematic review processes to aggregate findings from multiple studies in epidemiology.	[19]
Gemini Advanced	Predictive Analytics in Patient Care	Analyzes EHRs to predict patient health risks and outcomes.	Mayo Clinic employs Gemini Advanced to identify patients at risk of cardiac diseases.	[20]
	Genomic Data Interpretation	Supports genomic research by analyzing complex genetic information.	Used in oncology research to identify genetic markers relevant to personalized cancer treatment.	[21]
	Multi-Omics Data Integration	Integrates genomic, proteomic, and metabolomic data to uncover insights into disease mechanisms.	Applied in multi-omics studies to understand the complex interactions in diseases like diabetes.	[22]
Co-pilot	Clinical Workflow Optimization	Streamlines clinical documentation and administrative tasks.	Integrated with Microsoft Teams at St. Luke’s University Health Network for efficient documentation.	[23]
	Patient Engagement and Monitoring	Automates patient communication for appointment reminders, follow-ups, and health tips.	Deployed in healthcare systems for sending automated patient reminders and educational content.	[24]
	Data Analysis in Clinical Research	Aggregates and analyzes data from clinical trials to inform research findings.	Used in clinical trial data analysis to identify trends and outcomes across diverse patient populations.	[25]

Accelerating medical research through AI innovations

AI's role in advancing medical research is undeniable, offering support across fields like genomics, drug discovery, and epidemiology, thereby enriching the healthcare landscape. In genomics, AI, particularly through deep learning, supports researchers by sifting through genomic sequences to identify disease-predisposing mutations as illustrated in Table 2. This AI-assisted research aids in crafting personalized treatment plans that align with individual genetic profiles, enhancing treatment efficacy while minimizing side effects. It's a step towards personalized medicine, supplementing human expertise in unraveling complex genetic disorders such as cancer and Alzheimer's [10].

In drug discovery, AI's predictive capabilities serve as a valuable tool for researchers, identifying promising therapeutic candidates swiftly compared to traditional methods. This accelerates the development of new drugs and reduces associated costs, supporting the pursuit of treatments for a wider range of diseases. However, it's crucial to note that AI acts as a supportive tool in this process, augmenting but not replacing the critical decision-making roles of human experts in determining the safety, efficacy, and ethical considerations of new treatments [9-11].

AI's utility extends to epidemiology, where it has played a supportive role in managing public health crises like the COVID-19 pandemic. AI models have assisted public health officials by analyzing vast datasets to track virus spread, predict outbreaks, and inform containment strategies. While AI provides valuable insights for public health decision-making, it's essential to recognize that these decisions ultimately rely on human judgment, considering patient and community values, priorities, and social dynamics [12].

This revision indicates AI's position as an auxiliary tool in medical research and healthcare, reinforcing the principle that patient-centered care fundamentally depends on human insight, empathy, and the consideration of individual patient needs and contexts.

Challenges in health sciences

Addressing Complex Challenges in Health Sciences With AI Integration

The integration of AI within healthcare systems introduces multifaceted challenges, particularly concerning data privacy and security as seen in Table 3. The reliance on AI for diagnostics, treatment planning, and patient monitoring has led to an increase in the volume of sensitive data processed by AI algorithms, raising the stakes for data breaches and unauthorized access [13].

**Table 3:** Challenges in health sciences and AI integration Challenge: Identifies the specific obstacle or issue encountered in integrating AI technologies within healthcare systems. Description: Provides a brief explanation of the challenge, outlining its nature and scope. Impact on Healthcare: Discusses how the challenge affects various aspects of healthcare, including patient care, system efficiency, ethical considerations, and overall healthcare delivery. Real Study: Cites a relevant research study or publication that addresses or highlights the challenge. This includes the authors, the title of the work, and publication details, offering readers a resource for further exploration of the topic.

Challenge	Description	Impact on Healthcare	Reference
Data Privacy and Security	Concerns about protecting sensitive patient information within AI systems.	Compromises patient confidentiality and trust.	[14]
Interoperability and System Integration	Difficulty integrating diverse AI technologies with existing healthcare infrastructures.	Limits the effectiveness of AI applications in clinical settings.	[15]
Algorithmic Bias and Fairness	Risk of AI algorithms perpetuating biases based on training data.	Leads to unequal treatment outcomes and healthcare disparities.	[16]
Ethical and Legal Considerations	Navigating ethical and legal implications of AI decisions in patient care.	Raises concerns about responsibility and consent in AI use.	[17]
Resistance to Adoption	Skepticism among healthcare professionals and patients towards AI.	Hinders widespread adoption and optimization of AI in healthcare.	[18]
Resource Limitations	Financial, technological, and human resource constraints in AI implementation.	Limits access to AI benefits, especially in low-resource settings.	[19]

Implementing Advanced Cybersecurity Protocols

To counteract these risks, it's imperative for healthcare organizations to adopt comprehensive cybersecurity protocols. This involves the integration of encryption technologies to protect data both at rest and in transit, the deployment of advanced intrusion detection systems to monitor and respond to suspicious activities promptly, and the establishment of secure data storage infrastructures. Furthermore, AI algorithms need to be inherently secure, incorporating features such as differential privacy to ensure that while valuable insights are derived, individual data points remain confidential.

Upholding Regulatory Compliance and Patient Trust

Adherence to stringent regulatory standards such as the GDPR and HIPAA is critical, not only to meet legal requirements but also to foster patient trust. These regulations mandate precise guidelines on data handling, patient consent, and rights, urging healthcare entities to establish comprehensive data governance frameworks. To ensure regulatory compliance, routine audits, extensive staff training on data protection norms, and the establishment of AI-aligned policies are essential [20].

Revamping Consent Management for the AI Era

The dynamics of consent management evolve in the context of AI, where patients must be fully informed about the AI's role in their healthcare, including data usage, potential benefits and risks, and the scope of data sharing. Transparent and patient-centric consent processes are crucial, enabled by digital platforms that offer clear, understandable consent forms and flexible consent preferences.

Prioritizing Anonymization and Data Minimization

Protecting privacy through anonymization of patient data before AI processing is paramount. Techniques like data masking and tokenization effectively obscure patient identities, significantly mitigating privacy risks. Adhering to the principle of data minimization ensures that only essential data is processed, aligning with specific healthcare objectives.

Future-Oriented Challenges and AI Evolution

Despite these preventative measures, the evolving nature of cyber threats and AI advancements present continuous challenges. The risk of sensitive data exposure through AI outputs, known as inference attacks, necessitates relentless research and development of mitigation strategies.

The growing interoperability among diverse healthcare systems and AI solutions further complicates the cybersecurity landscape, underscoring the need for a unified, sector-wide cybersecurity strategy.

Ensuring Seamless AI Integration Within Healthcare Ecosystems

The integration of AI into existing healthcare infrastructures requires a holistic approach that considers both technological and organizational dimensions. Effective change management strategies are vital, encompassing technical AI deployment and significant human elements such as workforce training, workflow optimization, and the cultivation of an innovation-driven organizational culture. These strategies should be customized to fit the unique requirements of each healthcare facility, considering its specific challenges, operational needs, and technological framework.

Compliance with interoperability standards is fundamental in facilitating the smooth communication between AI technologies and various healthcare IT components, ensuring a cohesive healthcare service delivery. Standards like HL7 and FHIR are instrumental in achieving this integration, allowing AI tools to seamlessly blend into and enhance the healthcare delivery ecosystem [21].

Maintaining AI Systems' Reliability and Precision

In the healthcare domain, where AI decisions have direct implications on patient care, the accuracy and reliability of AI systems are imperative. Establishing comprehensive governance frameworks to oversee AI systems' lifecycle is crucial in maintaining the expected high standards in healthcare.

Continuous validation and adaptation of AI systems are necessary to align with the evolving landscape of medical knowledge, research breakthroughs, and clinical practices. This includes rigorous testing for algorithmic biases, ensuring AI-generated recommendations are fair and applicable across diverse patient groups.

External certification and peer evaluations by medical professionals further solidify AI systems' reliability, providing an added layer of trust and ensuring AI applications contribute positively to healthcare outcomes [22-31].

Ethical considerations

Ethical Considerations in AI-Supported Healthcare

The ethical integration of AI in healthcare requires a balance between leveraging technological advancements and maintaining the primacy of human judgment, especially in ensuring equity, accountability, and respect for patient autonomy.

Complementing Human Judgment to Mitigate Bias

While AI systems can enhance healthcare delivery, they may also carry biases from their training datasets or inherent algorithmic tendencies. To mitigate these biases and prevent disparities in healthcare outcomes, it's crucial to assemble diverse datasets that accurately represent patient populations. Additionally, AI models must be continually monitored and adjusted by healthcare professionals to correct emergent biases, ensuring AI-supported services remain fair and inclusive [32].

Enhancing Accountability in AI-Supported Decisions

The complexity of AI algorithms can sometimes obscure decision-making processes, challenging the ethical delivery of healthcare. Establishing clear, transparent governance that documents and reviews AI-supported decisions is essential to maintaining trust and ensuring AI serves as an aid to human decision-makers, not a replacement. These accountability measures reinforce the role of healthcare professionals in interpreting AI suggestions within the broader context of patient care [33-36].

Evolving Informed Consent With AI

The introduction of AI into healthcare necessitates evolving the informed consent process to address the nuances of AI use. Consent protocols must clearly explain the role of AI in patient care, detailing how AI-supported decisions are made, the data used, and the associated risks and benefits. This ensures patients are fully informed and can participate actively in decisions about their care, with healthcare professionals guiding and interpreting AI outputs in alignment with each patient's unique needs and values [37-38]. By emphasizing AI's role as a supportive tool rather than a decision-maker, this revised section aims to clarify the ethical framework within which AI should be integrated into healthcare. It underscores the importance of human oversight in AI applications, ensuring technology enhances patient-centered care without compromising the personal touch and understanding that are hallmarks of the healthcare profession.

## Conclusions

The integration of AI in healthcare, exemplified by technologies such as ChatGPT, Gemini Advanced, and Co-pilot, heralds a transformative era for the sector. These AI models promise to enhance healthcare delivery by making it more accessible, personalized, and efficient, covering a broad spectrum from mental health support to diagnostic improvements. While the potential benefits are substantial, realizing them fully entails navigating challenges related to data privacy, interoperability, and ethical concerns. As we move forward, the collaborative effort of technologists, healthcare professionals, and policymakers will be crucial in harnessing AI's capabilities responsibly and effectively, ensuring that these advancements lead to tangible improvements in patient care and medical research.
